# A genome-wide association study identifies *Arabidopsis thaliana* genes that contribute to differences in the outcome of infection with two *Turnip mosaic potyvirus* strains that differ in their evolutionary history and degree of host specialization

**DOI:** 10.1093/ve/veab063

**Published:** 2021-06-30

**Authors:** Anamarija Butković, Rubén González, Mark Paul Selda Rivarez, Santiago F Elena

**Affiliations:** Instituto de Biología Integrativa de Sistemas (I2SysBio), CSIC-Universitat de València, Catedrático Agustín Escardino 9, Paterna, València 46980, Spain; Instituto de Biología Integrativa de Sistemas (I2SysBio), CSIC-Universitat de València, Catedrático Agustín Escardino 9, Paterna, València 46980, Spain; Instituto de Biología Integrativa de Sistemas (I2SysBio), CSIC-Universitat de València, Catedrático Agustín Escardino 9, Paterna, València 46980, Spain; Department of Biotechnology and Systems Biology, National Institute of Biology, Večna pot 111, Ljubljana, 1000, Slovenia; Instituto de Biología Integrativa de Sistemas (I2SysBio), CSIC-Universitat de València, Catedrático Agustín Escardino 9, Paterna, València 46980, Spain; The Santa Fe Institute, 1399 Hyde Park Road, Santa Fe, NM 87501, USA

**Keywords:** emerging viruses, GWAS, host-range, *Potyvirus*, specialism-generalism continuum, virus evolution, virus-host interactions

## Abstract

Viruses lie in a continuum between generalism and specialism depending on their ability to infect more or less hosts. While generalists are able to successfully infect a wide variety of hosts, specialists are limited to one or a few. Even though generalists seem to gain an advantage due to their wide host range, they usually pay a pleiotropic fitness cost within each host. On the contrary, a specialist has maximal fitness within its own host. A relevant yet poorly explored question is whether viruses differ in the way they interact with their hosts’ gene expression depending on their degree of specialization. Using a genome-wide association study approach, we have identified host genes whose expression depends on whether hosts were infected with more or less specialized viral strains. Four hundred fifty natural accessions of *Arabidopsis thaliana* were inoculated with *Turnip mosaic potyvirus* strains with different past evolutionary histories and that shown different degrees of specialization. Three disease-related traits were measured and associated with different sets of host genes for each strain. The genetic architectures of these traits differed among viral strains and, in the case of the more specialized virus, also varied along the duration of infection. While most of the mapped loci were strain specific, one shared locus was mapped for both strains, a disease-resistance TIR-NBS-LRR class protein. Likewise, only putative cysteine-rich receptor-like protein kinases were involved in all three traits. The impact on disease progress of 10 selected genes was validated by studying the infection phenotypes of loss-of-function mutant plants. Nine of these mutants have altered the disease progress and/or symptoms intensity between both strains. Compared to wild-type plants six had an effect on both viral strains, three had an effect only on the more specialized, and two were significant during infection with the less specialized.

## 1. Introduction

Viruses are constantly facing heterogeneity in the hosts they infect. They face species with different response to infection or in many instances differences in immune status among individuals within the same host species. Some viruses adapt to a particular host species, genotype, or even cell type in which they efficiently complete their reproductive cycle (Turner and Elena 2000; Cooper and Scott 2001; Cuevas, Moya, and Elena 2003; Bedhomme, Lafforgue, and Elena 2012; Hillung et al. 2014; Navarro et al. 2020). These viruses are called specialists. Specialist viruses pose a great threat e.g. to monocultured crops since well-adapted viruses usually show enhanced within-host replication rates that are often associated with stronger symptoms (Roossinck 2010; Lacroix et al. 2014; Stobbe and Roossinck 2016). Examples of specialist viruses are *Dengue flavivirus* and *Mumps orthorubulavirus*, among mammalian viruses, and *Barley stripe hordeovirus* from plants (Elena, Agudelo-Romero, and Lalić 2009; Roossinck 2010). Other viruses infect hosts from widely different cell types, genotypes, species, or even higher taxonomical units and are dubbed generalists (Elena, Agudelo-Romero, and Lalić 2009). *Cucumber mosaic cucumovirus* (that infects more than 1,000 plant species) and the *Alphainfluenzavirus* (that infects birds, humans, and other mammalian species) are examples of generalist viruses (Elena, Agudelo-Romero, and Lalić 2009).

Each host range strategy comes with advantages and disadvantages. By hyperspecializing in a single host, a virus can limit interspecific competition and better access limited resources (Elena, Agudelo-Romero, and Lalić 2009; Bedhomme, Hillung, and Elena 2015). The advantage of generalism is the successful infection of multiple hosts. However, there is an obvious limitation to generalism: by being able to infect multiple hosts a virus does not maximize fitness in any particular one, conforming to the *jack-of-all-trades is a master of none* hypothesis (Whitlock 1996; Bedhomme, Hillung, and Elena 2015). It is proposed that selection favors specialist viruses because there is a trade-off limiting the fitness of a generalist virus in any of the alternative hosts and evolution proceeds faster in narrower niches (Woolhouse 2001). Antagonistic pleiotropy, where beneficial adaptations to a particular host could be disadvantageous in another (Lalić, Cuevas, and Elena 2011), is the most commonly claimed mechanism to explain this trade-off. Furthermore, to infect multiple hosts, viruses might need to encode for additional genetic information that would slow down their replication and increase their mutational fragility. Also, mutations that are fixed in order to compensate for antagonistic pleiotropy limit access to alternative evolutionary paths towards global maxima in the fitness landscape, reducing evolvability (Cervera, Lalić, and Elena 2016). All these characteristics make specialists capable of faster evolution and adaptation than generalists in the face of perturbations or new environments (Bedhomme, Hillung, and Elena 2015; Bono, Draghi, and Turner 2020). Although specialists tend to adapt faster to single hosts, generalists usually outcompete them in fluctuating environments by being more prepared to survive and reproduce as a consequence of having similar fitness in different hosts (Kassen 2002; Dennehy et al. 2013). This allows generalist viruses to have higher initial fitness compared to specialists when infecting novel host species, making them most likely emerging and re-emerging pathogens (Woolhouse and Gowtage-Sequeria 2005; Turner et al. 2010). Indeed, this theory has widespread support by experiments in which viral lineages being sequentially exposed to different hosts for long periods of time maximize their fitness in all hosts to the same extent as the corresponding specialist, thus overcoming the expected costs of generalism (Turner and Elena 2000; Duffy, Turner, and Burch 2006; Deardorff et al. 2011; Bedhomme, Lafforgue, and Elena 2012; Remold 2012).

Before moving forward, we would make a remark about definitions: specialist and generalists are usually defined by many authors as the two sides of a coin, as discrete events. That is, a virus would be defined as a specialist if and only if it infects one or, at best, a few closely related host species (or genotypes). In contraposition, a virus would be a generalist if and only if it infects more than one host species (or genotypes). This binary definition, however, does not fully reflect the complexity of interactions between parasites and their potential hosts. Specialism and generalism are broad terms that can be applied to different levels; viruses that are able to infect only one or more different hosts species, cell types, or genotypes (Turner and Elena 2000; Cooper and Scott 2001; Cuevas, Moya, and Elena 2003; Bedhomme, Lafforgue, and Elena 2012; Navarro et al. 2020). Let us take a very simple numerical example that easily illustrates this inconsistency. Imagine that a viral strain infects 100 different hosts (species, genotypes, or cell types) but, in 99 of them, produces few, say 10, new viral particles, whilst, in the remaining one host, it produces 10^10^ new viral particles. Now imagine a second viral strain that infects the same 100 different hosts but produces ∼10^5^ new viral particles in all of them. According to the binary definition, both strains must be considered as generalists. However, in our view the first strain is much closer to the specialist end while the second strain is much closer to the generalist end of a continuum of possible interactions. In this study, we would take this second definition and refer to specialist and generalist (or less specialist) viral strains of TuMV not on the basis of whether they infect one or more host genotypes but on the basis of *how well* they do across accessions.

The genetic basis of the observed differences between generalist and specialist viruses is actually poorly understood, at least from the perspective of the interaction of these two strategies with the host’s gene expression. Differences between the genomes of generalist and specialist viruses have been previously described (Takeuchi et al. 1991; Llamas-Saiz et al. 1996; Remold, Rambaut, and Turner 2008; Deardroff et al. 2011; Hillung et al. 2014; Navarro et al. 2020). However, so far just one study has sought to explore differential host responses associated with each virus strategy (Hillung et al. 2016).

Here, we aim to explore whether viral strains with different degrees of specialization affect the plant physiology and disease progress in different ways, identifying differentially responding candidate host genes. To reach this goal, we have undertaken a genome-wide association study (GWAS) approach. GWAS has gained popularity over the last 20 years due to the increasing number of genome sequences available for a wide range of organisms (Cantor, Lange, and Sinsheimer 2010; Bush and Moore 2012). The basis of GWAS is capturing single-nucleotide polymorphisms (SNPs) along the genome of an organism and using statistical methods (such as linear mixed models) to infer the association of SNPs with the trait being analyzed. The common disease-common variant hypothesis posits that common interacting alleles at multiple disease-predisposing loci underlie the most common diseases (Bush and Moore 2012). This hypothesis would justify the use of GWAS in the identification of alleles associated with specific phenotypes. This connection permits the identification of genetic risk factors for disease, such as susceptibility and resistance to viral infections (Korte and Farlow 2013). One of the most relevant inferences from GWAS is trait heritability, which indicates how much of the observed phenotypic variation is explained by genotypic variation (SNPs) relative to the contribution of environmental factors (Zaitlen and Kraft 2012).

Identifying host factors responsible for resistance or permissiveness to infection is a major goal when studying host–pathogen interactions, as this knowledge will help in better management of diseases. Here, we have characterized the infection of two strains of turnip mosaic virus (TuMV; species *Turnip mosaic potyvirus*, genus *Potyvirus*, Family *Potyviridae*) that differ in their degree of specialization in 450 natural accessions of *A.thaliana* (L.) Heynh. TuMV infects mostly *Brassicaceae* and is widespread worldwide causing important economical loses by damaging several important crops (Ohshima et al. 2002; Yasaka et al. 2017). One of the natural hosts of TuMV is *A. thaliana* (Pagán et al. 2010). This plant is undoubtedly one of the most suitable organisms for GWAS. It has over 1,000 natural accessions genotyped and described so far from Eurasia, North America, and North Africa (1001 Genomes Consortium 2016). Genotypes can be maintained by self-fertilization for an unlimited number of generations, facilitating GWAS and making phenotyping highly reproducible (Korte and Farlow 2013). The viral strains used in this study were obtained by Navarro et al. (2020) after experimental evolution of an *A. thaliana*-naïve ancestral TuMV isolate. This ancestral TuMV was evolved in plant genotypes deficient in different disease signaling pathways or in the presence of recessive susceptibility genes, resulting in two particular strains that largely differed in their experimental degree of specialization (see Section 2.2 below for details on the evolutionary history of these two strains).

In summary, the response to infection of 450 *A. thaliana* natural accessions from different geographic regions was phenotyped in a controlled common garden setting. These accessions were inoculated with two TuMV strains that differ in their degree of specialization. Infection data were analyzed using GWAS, specifically looking for SNPs differentially associated with infection with each strain. The genetic architecture of the phenotyped disease-related traits was also studied using the Bayesian sparse linear mixed model (BSLMM).

## 2. Materials and methods

### 2.1 Plant material and growth conditions

Four hundred and fifty *A. thaliana* accessions (Supplementary File S1) from the 1,001 *Arabidopsis* genome collection (https://1001genomes.org last accessed 29 June 2021; 1001 Genomes Consortium 2016) were phenotyped. The accessions were representative of the global species distribution. To ensure all the accessions were at a similar growth stage and to reduce the noise that large differences in vegetative development could cause, we confirmed that all selected accessions reached growth stage 3.2–3.5 in Boyes et al. (2001) scale ∼21 days after germination in our experimental growth conditions [16 h day/8 h night with temperature of 24°C day/20°C night, 45 per cent relative humidity and 125 µmol m^−2^s^−1^ of light intensity (1:3 mixture of 450 nm blue and 670 nm purple light-emitting diode)].

### 2.2 Virus inoculum and inoculation procedure

The two strains of TuMV used in this study were obtained after 12 passages of experimental evolution in mutant genotypes of the *A. thaliana* accession Col-0, as detailed in Navarro et al. (2020). Among all the resulting viral lineages, lineage L4 evolved in the *enhanced disease susceptibility 8* (*eds8-1*) mutant, hereafter referred as TuMV-G, and lineage L4 evolved in the *jasmonate insensitive 1* (*jin1*) mutant, referred as TuMV-S, showed strikingly different host ranges. The *eds8-1* plants lacked the EDS8 protein, causing the reduction of the expression of plant defensin genes and reduced induced systemic resistance but enhanced systemic acquired resistance (SAR). The *jin1* plants lacked the JIN1 protein, causing the loss of jasmonic acid (JA) signaling that is a negative regulator of salicylic acid (SA)-dependent signaling. This results in a constitutive expression of SAR. The *eds8-1* plants turned out to be the most resistant ones to TuMV infection, while the *jin1* plants were the most susceptible ones. TuMV-G was able to infect all plant genotypes tested by Navarro et al. (2020) with equal fitness, while TuMV-S infected only *jin1* well. Indeed, Navarro et al. (2020) calculated Blüthgen’s *d*’ specialization indexes (Blüthgen, Menzel, and Blüthgen 2006) for these two strains, finding that TuMV-G had *d*’ = 0 (no specialization) while TuMV-S had *d*’ = 1 (complete specialization). In agreement with previous potyvirus-*A. thaliana* studies (Hillung et al. 2014; González, Butković, and Elena 2019), more permissive hosts (herein *jin1*) were selected for specialist viruses while more restrictive hosts (in this case, *eds8-1*) were selected for generalist viruses. At the genomic level, TuMV-G and -S differed in a total of seven point mutations (Navarro et al. 2020). Relative to the ancestral naïve TuMV strain, TuMV-G contains three nonsynonymous mutations, all affecting the VPg protein (H33Y, D113N, and K121E). Likewise, TuVM-S has two synonymous mutations (*HC-Pro*/C1760U and *P3*/U3269C) and two nonsynonymous ones (VPg/R118H and CP/S70N). Therefore, TuMV-S and -G lie at two different locations in the specialist—generalist continuum. While TuMV-S behaves more like a specialist, showing high fitness only in its local host genotype, TuMV-G would better be described as a generalist that infects and induces similar disease severity across multiple host genotypes.

TuMV-G and -S infected plant tissues were frozen in liquid N_2_ and homogenized and mixed with 10 volumes of inoculation buffer (50 mM KH_2_PO_4_ pH 7.0, 3 per cent polyethylene glycol 6,000, 10 per cent carborundum) right before the mechanical inoculations. The two TuMV strains were mechanically inoculated into healthy *A. thaliana* plants that were between 21 and 25 days old. The inoculation started from the plants that were the largest (8–12 leaves) giving the smaller plants extra time to grow, so all the accessions got inoculated at a similar size (Boyes’ 3.2–3.5). Three middle sized leaves were mechanically inoculated with 5 μl of infectious sap prepared in inoculation buffer. To further minimize differences due to inoculation efficiency all the inoculations were done by the same researcher. Hence, we assume that the inoculation failure rate would be the same among all accessions.

Eight plants per accession for each TuMV strain were inoculated, resulting in a total of 16 plants phenotyped and two mock-inoculated control plants per accession. Accessions were split into two blocks because of chamber space and workforce capacity. The inoculation procedure took about 3–4 days per block, where on consecutive days different accessions underwent the inoculation procedure because (1) it was not possible to inoculate all the plants on the same day due to the sheer number of them and (2) this way all the plants got synchronized in size at the moment of inoculation. The first block was inoculated from 6 May 2019 to 8 May 2019 and the second block was inoculated from 11 September 2019 to 14 September 2019. Three hundred and one hundred and fifty accessions were inoculated in each block. Pot trays contained four accessions inoculated with each viral strain along with their corresponding mock-inoculated controls. To reduce spatial correlations due the relative position of plants in the growth chamber, pots trays were translocated to a new random position everyday.

Col-0 loss-of-function (LOF) mutant genotypes that were used to confirm GWAS results (Supplementary File S2) were seeded on 3 June 2020 and inoculated, as described above, with the two TuMV strains on 23 June 2020. All LOF mutants and wild-type (WT) control plants were analyzed in one block in the same growth chamber with 10 plants per virus and per genotype and two mock-inoculated controls per combination.

### 2.3 Phenotyping

**Figure 1.**
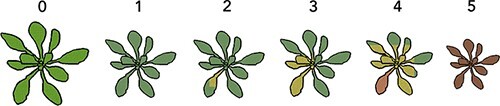
Symptoms scale that was used to evaluate the severity of symptoms in the plants during the 21 days period post inoculation. 0: no symptoms or healthy plant, 1: mild symptoms without chlorosis, 2: chlorosis is visible, 3: advanced chlorosis, 4: strong chlorotic symptoms and beginning of necrosis, and 5: clear necrosis and death of the plant.

Three phenotypic traits were measured (Supplementary File S3): (1) symptoms severity: on a scale from 0 to 5 (Fig. 1) measured at intermediate (14 days post-inoculation—dpi) and late (21 dpi) infection times so as to explore time-dependent differences in gene expression. Therefore, the status of plant infection was assessed by visual inspection for symptoms. Out of 450 accessions only two had less than 50 per cent of symptomatic plants, making the symptom development a good indication of virus infection. All the infected plants showed clear symptomatology associated with TuMV infection. The intensity of symptoms was also visually quantified, as the degree of damage TuMV causes correlates with its detrimental effects on the host. TuMV is a sterilizing agent, which directly impacts host reproduction (Vijayan et al. 2017). For a plant, the intensity of symptoms is an evolutionarily relevant trait, since the degree of damage on the vegetative organs and fruit development directly impacts its fitness (Pagan et al. 2007; Vijayan et al. 2017). (2) Infectivity: number of infected plants out of the total number of inoculated plants after 21 dpi. (3) Disease progress, calculated as the area under the disease progress stairs (*AUDPS*) (Simko and Piepho 2012). This measures the number of infected plants from the total number of inoculated plants each day during 21 days and combines it into a single value.

In the characterization of LOF mutant response to infection, *AUDPS* and symptoms intensity progression step (*AUSIPS*) curve (Kone et al. 2017) were measured. *AUSIPS* is calculated similarly to *AUDPS* and it summarizes the progression of the symptomatology through time. Every day during 21 days the severity of symptoms (scale shown in Fig. 1) was measured for all plants from each LOF mutant.

### 2.4 Genome-wide association mapping

Association analyses were done with a Python program based on LIMIX (Lippert et al. 2014) written by Prof. Magnus Nordborg’s group. LIMIX is a linear mixed model (LMM) that was used for single-trait analysis where SNPs and covariates were treated as fixed effects while the population structure and noise were treated as random effects. The kinship matrix (identical-by-state, IBS matrix) and the genotype data come from the 1,001 Genome project in *A. thaliana* (1001 Genomes Consortium 2016), consisting of the SNPs for the 1,135 genome accessions plus imputed SNPs of a set of accessions that were genotyped with a 250k SNP chip. Kinship measures the degree of genetic relatedness between individuals and is used to remove confounding factors that decrease power and increase the false positive rate in GWAS.

Data were explored with SPSS version 25 (IBM Corp., Armonk NY, USA) and deviations between the phenotypic values were observed between the two blocks, therefore the block effect was accounted for in the GWAS analysis through the covariates option in LIMIX. Untransformed phenotypic data was used in the GWAS, since transformed phenotypic data did not show much improvement compared with the untransformed data. The untransformed *AUDPS* and infectivity distributions were characterized by significantly negative skewness (in all cases, *g*_3_ ≤ −1.378 ±0.082, *P* < 0.001) and leptokurtosis (*i.e.* fatter tails than the Normal; in all cases, *g*_4_ ≥ 1.907 ±0.163, *P* < 0.001). In the case of symptoms severity, the distributions were significantly skewed toward positive values (in all cases, *g*_3_ ≥ 0.631 ±0.082, *P* < 0.001) but significantly leptokurtic only at 14 dpi (*g*_4_ = 2.253 ±0.163, *P* < 0.0001). In all cases, a one-sample Kolmogorov-Smirnov test rejected the null hypothesis of Normal distribution (in all cases *D* ≥ 0.112, *P* < 0.001).

Out of ∼10 million SNPs (Seren 2018), 1,815,154 had a minor allele frequency higher than 0.05 for all phenotypes. To minimize false positives due to multiple testing (type I errors), we used the false discovery rate (FDR) or the −log*P* ≥ 5 threshold, whatever value was more conservative. FDR was calculated using the fdrBH function (with *q* = 0.001) of the mSTEM R package version 1.0. The exact FDR values used were as follows: for TuMV-G *AUDPS* 21 dpi FDR = 2.73 × 10^−10^, infectivity 14 dpi FDR = 6.32 × 10^−13^ and 21 dpi FDR = 1.78 × 10^−8^, and symptoms 14 dpi FDR = 9.59 × 10^−10^. While for TuMV-S it was calculated only for symptoms at 14 dpi FDR = 1.49 × 10^−12^ and 21 dpi FDR = 1.15 × 10^−9^. Manhattan and quantile–quantile (QQ) plots were drawn using rMVP R package (Yin et al. 2020).

Each significant SNP was tested for linkage disequilibrium (LD) within a 10 kb window by calculating *r*^2^ with the help of PLINK 1.9 (www.cog-genomics.org/plink/1.9/). Furthermore, the *r^2^* results were examined for indications of any SNPs in strong LD with other significant SNP outside of the region of the significant gene. A 10 kb window was taken because in *A. thaliana* LD decays rapidly within 10 kb (Kim et al. 2007; Gan et al. 2011).

### 2.5 Bayesian sparse LMM

To determine whether many variants with small effects or a small number of large effects (sparse) variants were contributing to the disease-related traits variability, the BSLMM method implemented in GEMMA was used to infer the genetic architecture of the measured phenotypic traits (Zhou and Stephens 2012; Zhou, Carbonetto, and Stephens 2013). BSLMM models the genetic contribution as the sum of a sparse component and a highly polygenic component. The proportion of genetic variance explained by sparse effects is represented by the parameter *PGE* ∈ [0, 1]. The second parameter in the model is the total variance explained (*PVE* ∈ [0, 1]) by additive genetic variants. *PVE* is a flexible Bayesian equivalent of the narrow sense heritability (*h*^2^) estimated by more classical linear mixed models (LMMs). Raw values were used for symptoms severity (discrete variable), while *AUDPS* and infectivity (continuous variable) were normalized by block using a univariate general linear model in SPSS. The block effect on the phenotypes was not incorporated as a covariate in GEMMA, as it was done in LIMIX, because the optimization algorithm in GEMMA causes errors if some covariates are identical for some genotypes. In all cases, Markov chain Monte Carlo (MCMC) were run with the default settings (burn-in at 100,000, sampling steps at 1,000,000, and recording every 10 steps) and minor allele frequency cut-off set at 5 per cent.

The posterior inclusion probability (*PIP*) for an SNP is the probability of including this SNP as causal in the MCMC analysis, estimated from posterior samples of a gamma distribution that reflects the sparse effects (Schaid, Chen, and Larson 2018). *PIP* can be used as a measure of the strength of the association that an SNP has with the corresponding phenotype. Variants with a large effect in at least 25 per cent of the MCMC samples were diagnosed as significant (*PIP* ≥ 0.25).

### 2.6 Validation of GWAS associations

Ten genes identified with the GWAS were selected for further study of their LOF effect on disease progress (Supplementary File S2). The 10 chosen Col-0 T-DNA insertion LOF mutants were selected on the criteria that (1) a candidate gene per each of the phenotypic traits per virus was included and (2) they were available as homozygous lines in Nottingham Arabidopsis stock center (NASC) (https://arabidopsis.info/BrowsePage last accessed 29 June 2021). *AUDPS* and the *AUSIPS* were calculated using the number of infected plants, and their symptomatology was measured during 21 dpi for each individual plant. For statistical comparisons, a bootstrap approach was taken. One thousand pseudo-replicated matrices, of equal dimensions to the original one (rows representing individual plants and columns representing dpi), were generated per experimental condition. The matrix rows were replaced, and thus, the temporal correlations across time points were preserved. This algorithm, implemented in R, generated kernel distributions for *AUDPS* and *AUSIPS*. The 89 per cent highest density intervals (HDIs) were calculated using the bayestestR R package in (Makowski, Ben-Shachar, and Lüdecke 2019).

R version 3.6.1 (Core Team 2020) in RStudio version 1.2.1335 (RStudio Inc., Boston MA, USA) was used for all the analyses mentioned in the previous paragraphs.

## 3. Results

### 3.1 Characterization of infection traits in natural accessions

**Figure 2.**
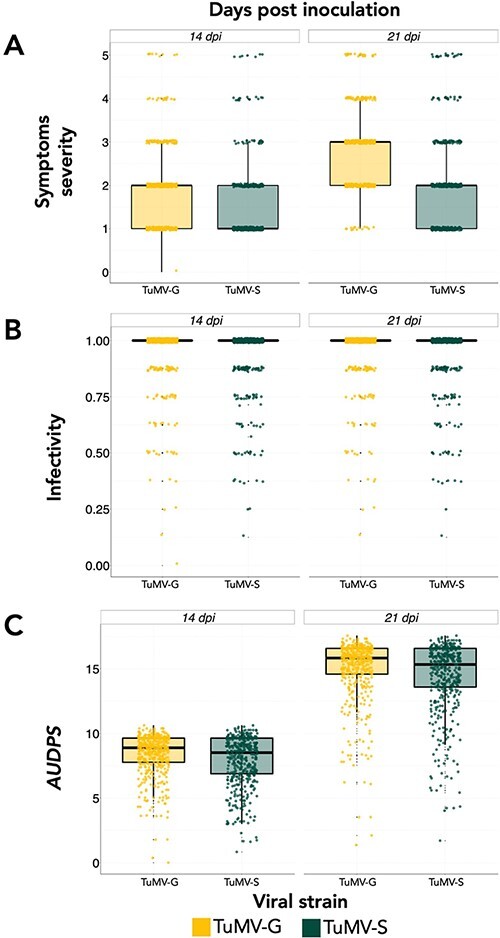
Distribution of the three disease-related traits characterized for each viral strain (TuMV-G in yellow and TuMV-S in green) infecting the 450 *A. thaliana* natural accessions at 14 (left) and 21 dpi (right). (A) Severity of symptoms, (B) infectivity, and (C) *AUDPS.*

The 450 *A. thaliana* accessions (Supplementary File S1) infected with the two TuMV strains varying in their degree of specialization were phenotyped for disease-related traits. Three disease-related traits were characterized by visual inspection and are shown in Fig. 2 (raw data provided in Supplementary File S3). Table 1 shows the results of the Scheirer–Ray–Hare test used to evaluate the effect of virus genotype, dpi, and their interaction on each disease phenotype. First, highly significant differences exist between the two viruses, with TuMV-G showing larger median values than TuMV-S for the three traits (median ±IQR for TuMV-G *vs* TuMV-S, respectively: symptoms severity 2.354 ±3.000 *vs* 1.779 ±4.000; *AUDPS* 11.692 ±8.822 *vs* 11.162 ±9.447; and infectivity 0.950 ±0.000 *vs* 0.936 ±0.000) (Fig. 2). Second, a highly significant effect has been observed associated with dpi for symptoms severity and *AUDPS* (median ±IQR at 14 *vs* 21 dpi, respectively: symptoms severity 1.721 ±4.000 *vs* 2.412 ±3.000; *AUDPS* 8.148 ±3.388 *vs* 14.708 ±3.625) but not for infectivity, indicating that the number of plants diagnosed as infected based on the presence of symptoms did not increase during the last seven days, while symptoms got more severe (Fig. 2). Third, a significant interaction between both factors has only been observed in the case of symptoms severity (Table 1), which in this case suggests that the difference between the two viral strains for this trait was larger at 21 dpi (relative change in means ∼40 per cent) than at 14 dpi (relative change in means ∼25 per cent) (Fig. 2).

**Table 1.** Non-parametric two-ways analysis of variance (Scheirer–Ray–Hare) test of the two main effects and their interaction for each of the three disease-related traits experimentally determined.

		Symptoms severity (*S*)		*AUDPS*		Infectivity (*i*)	
Source of variation	df	*H* ^a^	*P*	*H*	*P*	*H*	*P*
Virus genotype	1	191.414	<0.001	7.711	0.006	7.183	0.007
Dpi	1	250.766	<0.001	1,122.420	<0.001	0.257	0.612
Virus genotype by dpi	1	14.984	0.001	0.021	0.883	0.037	0.847

a *H* statistic follows a χ^2^ distribution.

Furthermore, it is well known that in the case of TuMV the set of *A. thaliana* genes differentially expressed changes along the stage of infection (Sánchez et al. 2015; Corrêa et al. 2020). Guided by these previous experiments, the infection traits were studied both at 14 and 21 dpi to account for potential differences between the viral strains at different stages. Accordingly, GWAS of the infection traits was performed at both time points.

### 3.2 Genetic architecture of disease-related traits

The 450 accessions accounted for 431,323 SNPs that were tested in both viral strains at both 14 and 21 dpi (Supplementary File S4) using the BSLMM analysis. This analysis evaluates how much of the observed *PVE* is explained by the genotyped SNPs and how important are the contributions of sparse effects to the *PGE*.

*AUDPS*, infectivity and symptoms severity had low *PVE* values (Supplementary File S4). The lowest *PVE* value was obtained for *AUDPS* [median 0.08 and 89 per cent HDI (0.03, 0.14)] and infectivity [median 0.08 and 89 per cent HDI (0.03, 0.13)] for TuMV-G at 14 dpi and for TuMV-S at 21 dpi [median 0.08 and 89 per cent HDI (0.03, 0.14)], while the largest value was obtained also for *AUDPS* measured at 14 dpi but for TuMV-S [median 0.28 and 89 per cent HDI (0.19, 0.38)]. In all other instances, *PVE* values were similar for both strains and between the two time points.

Regarding *PGE* (Supplementary File S4), on one hand, the smallest value was observed for the severity of symptoms induced by TuMV-G at 14 dpi [median 0.33 and 89 per cent HDI (0, 0.82)] and 21 dpi [median 0.33 and 89 per cent HDI (0, 0.81)] and by TuMV-S at 21 dpi [median 0.33 and 89 per cent HDI (0, 0.81)]. On the other hand, the largest *PGE* value was estimated for TuMV-G infectivity measured at 21 dpi [median 0.92 and 89 per cent HDI (0.71, 1)]. The percentage of *PVE* explained by large sparse effect variants (*PGE*) indicates that major effect loci account for between 50 and 90 per cent of PVE in *AUDPS* and infectivity traits, in both time points for both viruses (median values reported in Supplementary File S4). The number of variants with large effect size, the SNPs that explain most of the phenotype among the 431,323 SNPs, was low for infectivity and symptoms severity at 14 dpi for both viruses as well as for *AUDPS* and infectivity at 21 dpi also for both viruses (Supplementary File S4). To detect large-effect SNPs that might be contributing the most to the variance in disease-related phenotypes, a *PIP* ≥ 0.25 threshold was imposed in the BSLMM model in GEMMA. With this constrain, three highly significant SNPs have been detected. The first was detected for TuMV-S *AUDPS* estimated at 21 dpi. This SNP was mapped within the gene encoding for *AT2G04440*, a MutT/Nudix family protein (Supplementary Fig. S1). The second significant SNP was found for TuMV-G infectivity at 21 dpi within locus *AT3G19350* that corresponds to the gene *MATERNALLY EXPRESSED PAB C-TERMINAL* (*MPC*). The third significant SNP was also observed for TuMV-G infectivity at 21 dpi and corresponds to position 6,685,977 of an intergenic region on chromosome 3 (Supplementary Fig. S1). Chromosome 3 intergenic position 6,685,977 is between loci *AT3G19290*, which corresponds to the gene *ABA-responsive element binding protein4* (*ABF4*), and *AT3G19280*, which corresponds to the gene *fucosyltransferase11* (*FUT11*). Interestingly, the chromosome 3 intergenic position 6,685,977 shows a strong LD (*r*^2^ = 1; in a 10 kb window) with *FUT11*.

Next, we ran an LD analysis to discover SNPs at different loci that might be significantly associated. A total of four pairs of SNPs located at different loci showed significant LD values (*r*^2^ > 0.5 in all cases; Supplementary File S5). The rest of SNPs showed strong LD only with other SNPs within the same locus. All four significant pairs involved protein coding genes. Three of the four pairs of SNPs in LD were mapped for TuMV-S.

In summary, for this host-pathogen system, the genetic architecture of *AUDPS* and infectivity phenotypes is relatively simple, involving few small-effect SNPs along with one large effect SNP that is being responsible for the majority of variance in the observed phenotypes. Symptoms severity, however, is genetically more complex and involves many more small effect SNPs. For both viral strains, all the disease phenotypes have a similar genetic architecture between the two temporal stages (14 and 21 dpi).

### 3.3 GWAS identifies genetic loci associated with disease-related phenotypes differentially induced by virus strains that differ on their past evolutionary history and degree of specialization

**Figure 3.**
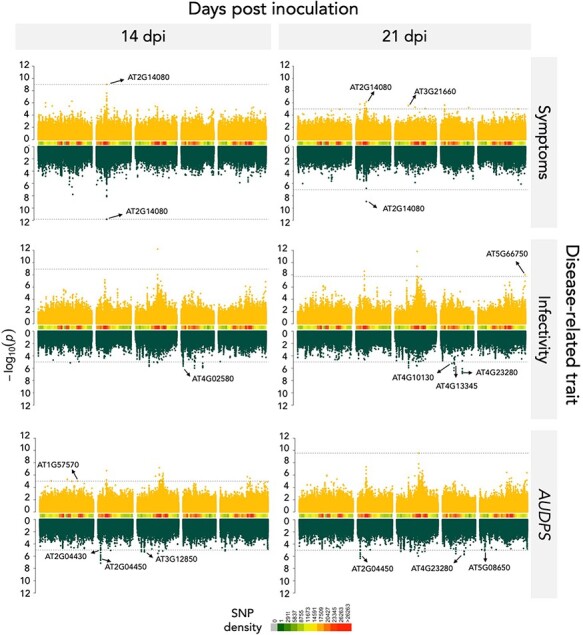
Manhattan plots of the analyzed disease-related traits. Data for TuMV-G are indicated in yellow and for TuMV-S in green. Peaks marked on the plots correspond to the most significant SNP values of the genes selected for the mutant analysis. SNP density shows how many SNPs are genotyped for a particular chromosomal region. The dashed lines indicate the significance threshold (FDR or −log*P* = 5 whenever computing FDR was not possible).

**Figure 4.**
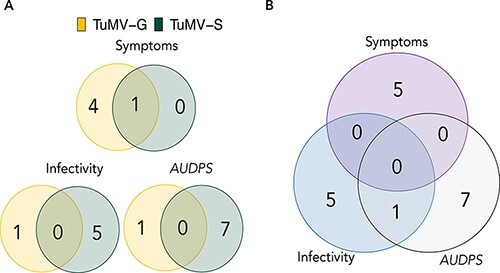
Venn diagram showing the number of unique and shared genes. (A) Genes mapped for each viral strain and disease-related traits. (B) Genes mapped for all disease-related traits pooling together both viral isolates.

The significantly associated SNPs for the three disease-related traits were visualized using Manhattan plots in (Fig. 3). The QQ-plots for infection traits showed no detectable population structure (Supplementary Fig. S2). Using the FDR or the −log*P* ≥ 5 thresholds determined for each of the traits (Supplementary File S5), a total of eight significant SNPs were identified for TuMV-G and 19 for TuMV-S infection (Supplementary File S5). Some of these SNPs were positioned within seven genes for TuMV-G and 12 for TuMV-S (Table 2 and Supplementary File S5). Most of the identified genes were unique to plants infected by TuMV-G or TuMV-S, with only one locus shared both at 14 and 21 dpi, for symptoms severity: *AT2G14080*, a TIR-NBS-LRR class family disease resistance protein (Fig. 4A).

**Table 2.** Significant genes detected using GWAS for the three disease-related traits during the course of infection with each viral strain.

Trait	dpi	Gene		Description	−log *P*
TuMV-G					
Symptoms severity	14 and 21	*AT2G14080*		Disease resistance protein (TIR-NBS-LRR class) family	9.02
Symptoms severity	21	*AT3G21660*	*PUX6*	Plant UBX domain-containing protein 6	5.54
Symptoms severity	21	*AT3G46590*	*TRP2*	Telomere repeat-binding protein 2	5.09
Symptoms severity	21	*AT4G04540*	*CRK39*	Putative cysteine-rich receptor-like protein kinase 39	5.65
Symptoms severity	21	*AT4G32660*	*AME3*	Serine/threonine-protein kinase AME3	5.22
*AUDPS*	14	*AT1G57570*	*JAL14*	Jacalin-related lectin 14	5.01
Infectivity	21	*AT5G66750*	*DDM1*	ATP-dependent DNA helicase DDM1	8.00
TuMV-S					
Symptoms severity	14 and 21	*AT2G14080*		Disease resistance protein (TIR-NBS-LRR class) family	11.83
*AUDPS*	21	*AT4G23280*	*CRK20*	Putative cysteine-rich receptor-like protein kinase 20	5.73
*AUDPS*	14 and 21	*AT2G04450*	*NUDX6*	Nudix hydrolase 6	6.45
*AUDPS*	14 and 21	*AT2G04440*		MutT/nudix family protein	6.11
*AUDPS*	21	*AT3G07470*		Transmembrane protein	5.20
*AUDPS*	14	*AT3G12850*		COP9 signalosome complex-related/CSN complex-like protein	5.18
*AUDPS*	14/21	*AT5G08650*	*CPLEPA*	Translation factor GUF1 homolog, promotes efficient protein synthesis in chloroplasts	5.14
*AUDPS*	14	*AT2G04430*	*NUDX5*	Nudix hydrolase 5	5.02
infectivity	14 and 21	*AT4G23280*	*CRK20*	Putative cysteine-rich receptor-like protein kinase 20	6.52
infectivity	14 and 21	*AT3G21980*	*CRRSP27*	Cystein-rich repeat secretory protein 27	
infectivity	14	*AT4G02580*		NADH dehydrogenase [ubiquinone] flavoprotein 2	5.64
infectivity	21	*AT4G13345*	*MEE55*	Serine-domain containing serine and sphingolipid biosynthesis protein	5.32
infectivity	21	*AT4G10130*	*T9A4.1*	DNAJ heat shock N-terminal domain-containing protein	5.22

Comparing the results at 14 and 21 dpi for TuMV-G, genes at 14 dpi seem more related to a general disease response whilst genes at 21 dpi are more specific and involved in ubiquitin-related processes. Such temporal difference is not seen for TuMV-S. This may suggest that plants responses to the less specialized strain change more dynamically than when infected with the more specialized one, in which case the response seems unchanged between the two time points studied. Fig. 4B shows that most of the identified genes (17) had an effect only in one of the disease-related traits. However, locus *AT4G23280* that encodes for the putative *cysteine-rich receptor-like protein kinase 20* (*CRK20*) was involved both in *AUDPS* and infectivity. Interestingly, locus *AT4G04540* mapped for symptoms severity also encodes for a putative *rich receptor-like protein kinase 39* (*CRK39*).

### 3.4 Experimental validation of identified genes

**Figure 5.**
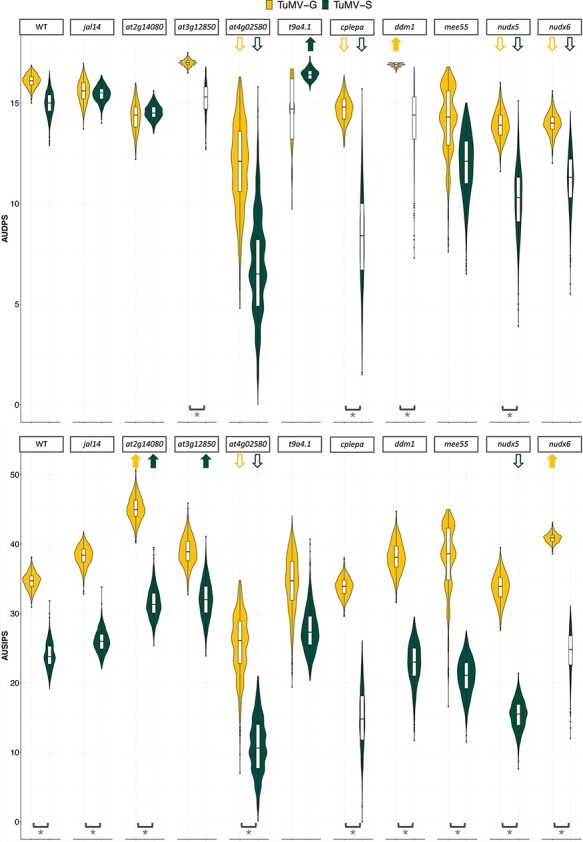
89 per cent HDI calculated for *AUDPS* and *AUSIPS* for each viral strain on each LOF mutant plant genotype. Not overlapping 89 per cent HDI between a given mutant and the WT plants is indicated by an arrow. Arrows pointing up indicate a significant positive difference in medians, while arrows pointing down indicate the opposite trend. Brackets and asterisks indicate significant differences between TuMV-G and TuMV-S disease progress or severity in the LOF mutant plant genotype being considered.

Ten of the identified genes were selected for a validation study in which the corresponding LOF mutants were inoculated with both viral strains and the disease progress was characterized (Fig. 5 and Supplementary File S6). Out of the 10 genes, one was shared between the two viral strains, two were unique for plants infected by TuMV-G and seven were unique for plants infected by TuMV-S. More genes were validated for TuMV-S because the GWAS mapped more significant SNPs upon infection with this strain. The selected LOF mutants were *jal14, nudx5, nudx6, at2g14080, at3g12850, t9a4.1, mee55, cplepa, ddm1*, and *at4g02580*. To evaluate differences in infection dynamics between the mutants and the WT plants, *AUDPS* and *AUSIPS* were calculated using the data collected along the 21 dpi. A comparation between the WT and LOF mutant values for each viral strain was done (Fig. 5, Supplementary File S6) based on the inferred 89 per cent HDIs. Differences in most of the LOFs were found when comparing the *AUDPS* values of the two viral strains with the WT (Supplementary File S6).

Evaluating the mutant *AUDPS* intervals, lower 89 per cent HDIs compared to the WT imply that these mutants have slower disease progress because the LOF gene is positively involved in the viral cycle and the virus uses it to aid its replication, translation, assembly, or movement. For the TuMV-S and TuMV-G infection, there are four mutants that have lower 89 per cent HDI compared to the WT: *at4g02580, cplepa, nudx5*, and *nudx6.*

Observing LOF mutants with *AUDPS* intervals higher than the WT suggests that the corresponding genes are involved in plant defense response against infection, removing them enhanced disease progress beyond the one observed for the WT plants. In TuMV-G infection, *ddm1* had 89 per cent HDI higher than the WT. In plants infected with TuMV-S only *t9a4.1* had higher 89 per cent HDI compared to the WT.

For *AUSIPS*, if mutant values had higher intervals than the WT it meant that the virus causes stronger symptoms in the absence of these host genes. This is the case for *at2g14080* and *nudx6* plants infected with TuMV-G. In the case of TuMV-S this happened in *at3g12850* and, as for TuMV-G, in *at2g14080* plants. An interesting observation was made for *nudx6* mutant for *AUDPS*, where it showed an opposite effect in comparation with *AUSIPS*.

Therefore, significant differences in *AUDPS* confidence intervals between WT and mutant plants confirm the role in infection of the genes that were knocked-out. When the mutant had lower *AUDPS* values (e.g. *at4g02580, cplepa, nudx5*, and *nudx6* for both viral strains), it confirmed the positive function of the gene in the viral replication. In contrast, mutants with values higher than those of the WT (*e.g. ddm1* mutants for TuMV-G and *t9a4.1* for TuMV-S) confirmed the role of the gene in the host defense. Comparations of *AUSIPS* values between WT and mutant plants also confirm the role of most of the studied genes in symptoms severity. The *at4g02580* plants had lower *AUSIPS* interval in the TuMV-G infection. Therefore, plants defective in an NADH-ubiquinone oxidoreductase susceptibility factor had a milder symptomatology than WT ones. Mutants *at4g02580* and *nudx5* also had lower *AUSIPS* intervals when infected with TuMV-S. Differences in symptoms severity progression were also significant between the two viral strains in the WT, *at2g14080, at4g02580, cplepa, ddm1, jal14, mee55, nudx5*, and *nudx6*. This difference indicates that the two viral strains cause different symptomatology in the WT and the majority of the mutants.

## 4. Discussion

Pathogens will have different virulence and induce different responses in their hosts depending on their adaptation history. For example, in the whole-genome transcriptomic study by Hillung et al. (2016), they compared the transcriptomic responses of six *A. thaliana* accessions infected with generalist or specialist strains of *Tobacco etch potyvirus*. They showed that the generalist virus manipulated a similar set of host genes across the experimental host range, while the specialist virus showed a more heterogeneous response. In our GWAS study, similar conclusions can be reached by comparing host genes associated with infection by the two TuMV strains differing in their degree of specialization. In the case of the less specialized strain, TuMV-G, fewer host candidate genes were identified compared to the more specialist strain, TuMV-S. This difference might have emerged as a consequence of the different evolutionary strategies of the two viruses or as a consequence of the limitations of the GWAS analysis, such as inability to identify all genetic determinants of complex traits that is a common problem in all similar methods. If we focus on the different evolutionary strategies of the two strains, we can say that selection has driven the less specialized (this is to say, the more generalist one) virus to manipulate a similar set of host genes across the host range for successful infection. In contrast, host-specific selective pressures modulated the evolution of the more specialized strain; hence, more genes associated with TuMV-S have been found by the GWAS analysis.

Mapped genes in the GWAS belonged to categories such as F-box proteins, kinase, hydrolase, LRR family proteins, disease resistance proteins, transcription factors, lectins, helicases, ubiquitin proteases, proteins involved in iron metabolism, pentatricopeptide repeat-containing, GTPases, and berberines, all of them being involved in the plant response to infection, the viral cycle or RNA metabolism (Ge and Xia 2008; Lee 2008; Corrêa et al. 2013; Manna 2015; Li et al. 2016; Guo et al. 2020; Herlihy, Long, and McDowell 2020; Huang et al. 2020). Locus *AT2G14080* was identified as significant for both viral strains in the analysis of symptoms severity. *AT2G14080* belongs to NBS-LRR genes that are the most numerous class of the *R* (resistance) genes in *A. thaliana*. Their effector recognition LRR domains recognize specific pathogens and can lead to a hypersensitive immune response (HR) or to an extreme resistance against the virus infection. An HR restricts the pathogen at the primary infection site causing cell death followed by SAR that increases SA accumulation and expression of pathogenesis-related genes (Meyers et al. 2003; Marone et al. 2013). There were also some strain-specific hits that were previously characterized as involved in plant defense or in some important part of the viral cycle. Indeed, host genes that differ between the two strains could be targets of differential selection during their experimental evolution. For example, ubiquitin protease and telomere repeat-binding protein 2 were specific responses of the plant to TuMV-G infection. While the Nudix hydrolase, NADH dehydrogenase and DNAJ heat shock proteins were specific for plants infected with TuMV-S strain.

Loci *AT4G04540* and *AT4G23280* both encode for cysteine-rich receptor-like protein kinases (*CRK39* and *CRK20*, respectively). *CRK*s are the only genes mapped in common for all three disease-related phenotypic traits. *CRK* genes are induced upon pathogen infection in *A. thaliana* via the SA signaling pathway, resulting in HR (Chen et al. 2004). In general, receptor-like protein kinases (RLKs), a large family with more than 600 members, are central players in the plant receptor kinase-mediated signaling involved in hormonal responses pathways, cell differentiation, plant growth and development, self-incompatibility, and symbiotic and pathogen recognition (Liang and Zhou 2018). Given their upstream role in the MAPK signaling cascades, it is not surprising that RLK expression has many pleiotropic effects on diverse plant phenotypes.

There was one gene that came up in the GWAS of both strains, *AT2G14080* that had a significant effect on the mutant involved with the two strains and it appears to be involved in plant defense. Two of the 10 genes selected for the mutant analysis came from TuMV-G analysis and seven came from the TuMV-S analysis. Eight of the selected host genes had a significant effect on the virus disease progress and/or symptoms *maternal effect embryo arrest 55* (*MEE55*, encodes for a serine and sphingolipid biosynthesis protein) and *AT1G57570* (*JAL14*, encodes for a member of the mannose-binding lectin superfamily protein) apparently had no significant effect on either viral strain under our experimental conditions. There were five host genes that had an effect in both viral strains: *AT2G14080, AT4G02580, CPLEPA, NUDX5*, and *NUDX6. AT2G14080* is an NBS-LRR resistance gene. These proteins monitor the status of plant proteins targeted by pathogens and activate a series of defense responses (McHale et al. 2006). By removing this host gene, viruses managed to induce stronger symptoms *AT4G02580* is a susceptibility factor and could aid viral pathogenesis (Kant et al. 2019). CpLEPA is a highly conserved chloroplastic translation factor that could assist viral transcription in the cytoplasm by enhancing the translation of chrolopastic proteins involved in photosynthesis to compensate for the negative side-effects of infection in chloroplasts activity (Ji et al. 2012; Li et al. 2013; Sanfaçon 2015). Mutants *nudx5* and *nudx6* are deficient in proteins that form part of the Nudix hydrolase family, which act as positive regulators in plant immunity (Ge and Xia 2008; Yoshimura and Shigeoka 2015), thus leading to a stronger anti-pathogen response. In both viral strains, they seem to have important roles for disease progress by enhancing viral replication or gene expression since viruses replicated worse when these two genes where knocked-out. Suggesting the possibility that these two Nudix hydrolases could have additional functions besides the one described in defense. This role for the two hydrolases in viral infection was not described before.

Genes that had an effect on the LOF mutant analysis for *AUDPS* for virus TuMV-G were *DDM1* and *T9A4.1*. Corrêa et al. (2020) showed that *ddm1* plants were more resistant to two different strains of TuMV. This might be because induction of SA-mediated defense in *ddm1* mutants may be an explanation of their resistance to TuMV. The opposite has been noticed for geminiviruses where *ddm1* mutant showed hypersusceptibility to infection (Raja et al. 2008). The reason for this was the methylation of viral genomes that is a plant defense mechanism; when methylation is reduced, plants are more susceptible (Raja et al. 2008). Differences in adaptation history of TuMV-G and the strains studied by Corrêa et al. (2020) might explain why TuMV-G replicates better in this mutant in our study. For TuMV-G, the lack of DDM1 might help the virus replicate better since defense genes are not properly methylated and henceforth their expression deregulated. Another significant gene in the mutant analysis was *T9A4.1*, which is involved in peptidyl-diphthamide biosynthetic processes and tRNA wobble uridine modification. Both of these processes are involved in translation modifications and this protein might have a role in an anti-pathogenic response.

For the strain TuMV-S in the *AUSIPS* values, one host gene had a significant effect on the mutant analysis, *AT3G12850*, which is involved in regulation of JA levels. Viruses infecting *at3g12850* plants replicate better. *AT3G12850*-encoded protein is a COP9 signalosome complex-related/CSN complex-like protein. The tomato yellow leaf curl Sardinia virus (TYLCSV) C2 protein interacts with CSN5 resulting in a reduction of JA levels. As previously shown, treating *A. thaliana* plants with exogenous JA disrupts TYLCSV infection (Lozano-Durán et al. 2011). It is known that plant viruses and herbivores have strategies to manipulate JA levels as this hormone confers defenses to the plant against biotic and abiotic stresses (Wu and Ye 2020). This means that in our pathosystem the JA is negatively affecting the viral replication.

A GWAS of TuMV infection in *A. thaliana* in a natural setting was recently performed by Rubio et al. (2019). None of the genes found by these authors were pinpointed in our study but this could simply reflect three major experimental differences: (1) Rubio et al. (2019) grew their plants in a natural setting where they were exposed to a changing environment. The highly complex natural setting can lead to much more heterogeneous gene regulations, as opposed to a controlled environment that minimizes external abiotic and biotic stressors. It has been shown before that abiotic stresses influence the response of the plant to viruses (Xu et al. 2008; Hily et al. 2016; González et al. 2021). Multiple stresses affecting the plant at the same time can be problematic when trying to identify genes responsible for the specific response of plants to virus infection. (2) The evolutionary histories of the TuMV strains used in both studies were largely different. While Rubio et al. (2019) used the UK1 isolate, we used strains derived from the YC5 isolate originally obtained from calla lily plants (Chen et al. 2003). (3) In our study, the 450 accessions were chosen to represent the world-wide genetic diversity of the species, while French accessions were largely overrepresented in Rubio et al. (2019) study. Rubio et al. (2019) identified six new genes above a threshold of −log*P* ≥ 4 in their GWAS analysis:*restriction to tobacco etch virus movement 3*, a DEAD box RNA helicase 1 candidate gene, *eukaryotic translation initiation factor 3b*, a protein with a pleckstrin homology domain, a protein containing a TIM barrel domain, and a key enzyme involved in the glutamate pathway. Our study identified 13 genes specifically mapped for viral infection response (Table 2), of which eight were experimentally confirmed as having roles in the plant response to TuMV-S and TuMV-G (Fig. 5). Despite the lack of matching genes mapped between both studies, there are similarities at the functional level: for both studies there were genes mapped that belonged to ATP-dependent DNA helicase, DnaJ domain superfamily protein and ubiquitin associated proteins.

Looking at the analysis of the underlying genetic architecture of each phenotyped trait, it was evident that some disease-related phenotypes were explained by few SNPs (infectivity and symptoms severity at 14 dpi for both viruses and *AUDPS* and infectivity at 21 dpi for both viruses as well), while some traits were highly polygenic and explained by a large number of SNPs (*AUDPS* for TuMV-S at 14 dpi). SNPs that passed the *PIP* threshold were mapped within locus *AT2G04440* (MutT/Nudix family protein) for *AUDPS* of TuMV-S at 21 dpi and position 6,685,977 in an intergenic region on chromosome 3, along with gene *MPC* for infectivity of TuMV-G at 21 dpi (Supplementary Fig. S1). All had possible roles in the viral infection. *AT2G04440* was previously characterized as an important player in the plant immune response (Ge and Xia 2008). *MPC* is an important translation initiation factor that binds to the viral VPg and the RNA-dependent RNA polymerase (RdRP) NIb of TuMV, affecting the viral RNA accumulation (Dufresne et al. 2008). The noncoding intergenic region at position 6,685,977 on chromosome 3 could be a promoter region involved in regulation of the expression of both *ABF4* and *FUT11. ABF4* controls the ABA-dependent stress response. It was previously shown that *Wheat yellow mosaic potyvirus* disturbs the ABA signaling pathway through the interaction between the viral RdRp and the wheat’s light-induced protein TaLIP thus facilitating virus infection (Zhang et al. 2019). There is no clear description of *FUT11* in plant virus infection, but it is involved in protein N-linked glycosylation and the intergenic position 6,685,977 shows a strong LD (*r*^2^ = 1; in a 10 kb window) with this gene.

Since the genome of *A. thaliana* is highly polygenic and is governed by small effect loci (as shown by the BSLMM analysis), our study might have missed some of the genes described in the literature as being involved in the potyvirus infection. Other explanation for the absence of previously described genes would be that they were not important in the context of our virus strains that were preadapted in specific mutants of *A. thaliana*.

Altogether, this work (1) describes differences between two strains whose past evolutionary history determined differences in their degree of specialization, (2) identifies and characterizes genes involved in the infection with more or less specialized viral strains, and (3) illustrates the variability of the genetic elements involved in a viral infection depending on the evolutionary history of the viral strain.

## Data availability

Supplementary data are available at GitHub https://github.com/sfelena/TuMV-specialist-generalist-GWAS, last accessed June 29th 2021.

## Supplementary Material

veab063_Supp
