# Supplementary figures and images for: A genome-wide association study identifies Arabidopsis thaliana genes that contribute to differences in the outcome of infection with two Turnip mosaic potyvirus strains that differ in their evolutionary history and degree of host specialization
Source: Virus Evol. 2021 Jun 30;7(2):veab063. doi: 10.1093/ve/veab063 (PMC8438913; doi:10.1093/ve/veab063)

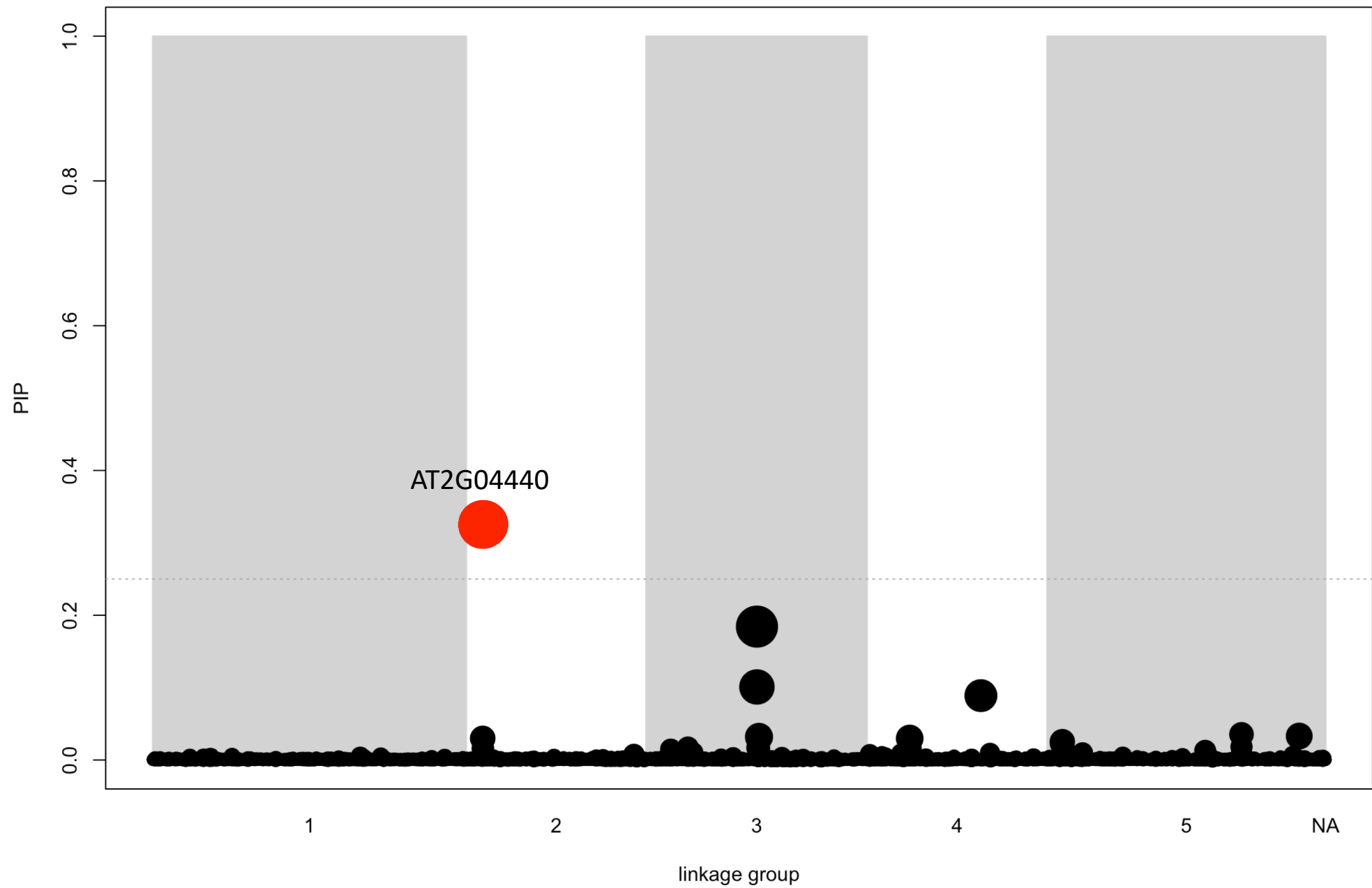

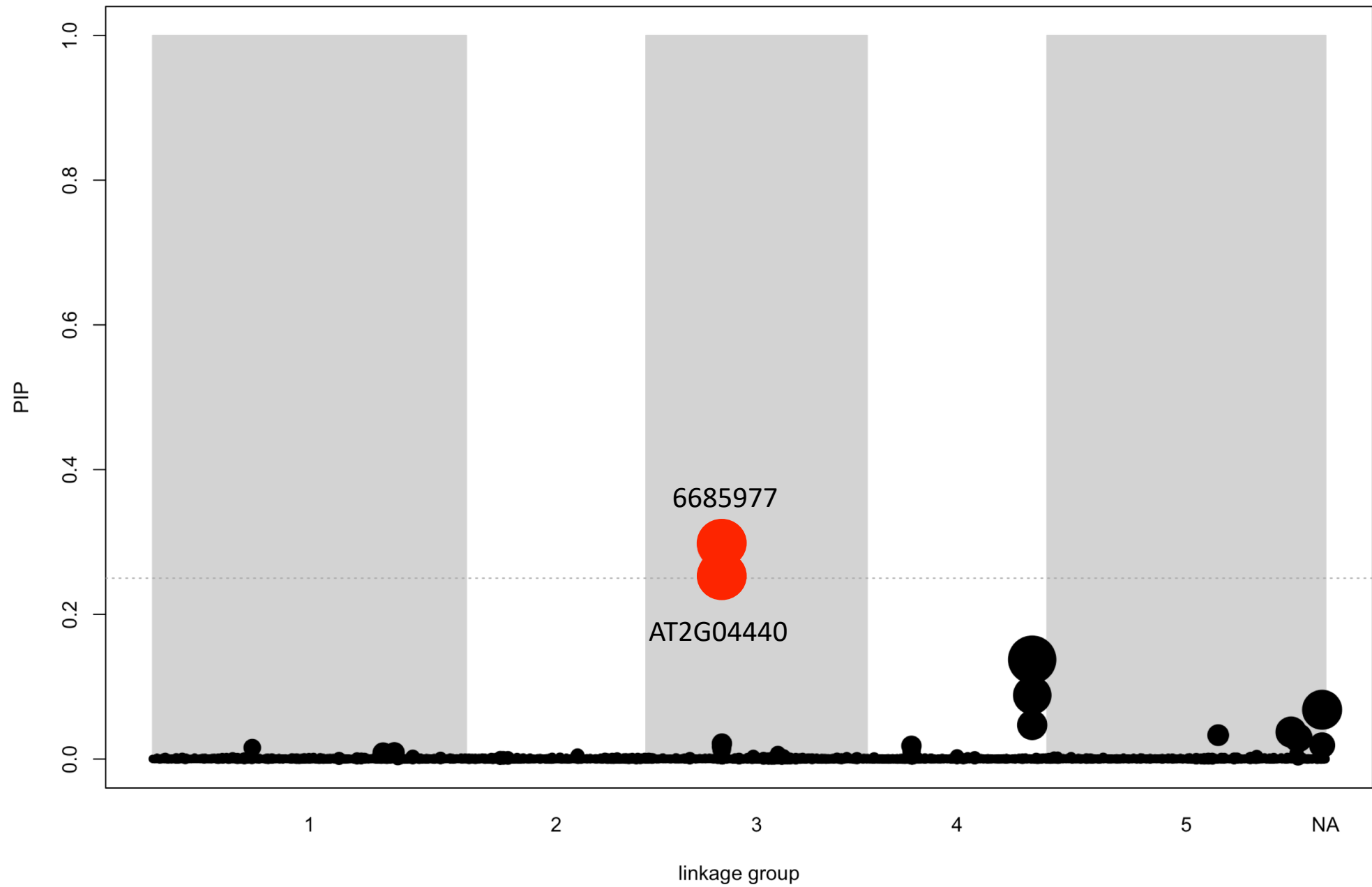

Supplement: veab063_Supp [file veab063_supp.zip › Supplementary Fig. S1.pdf]

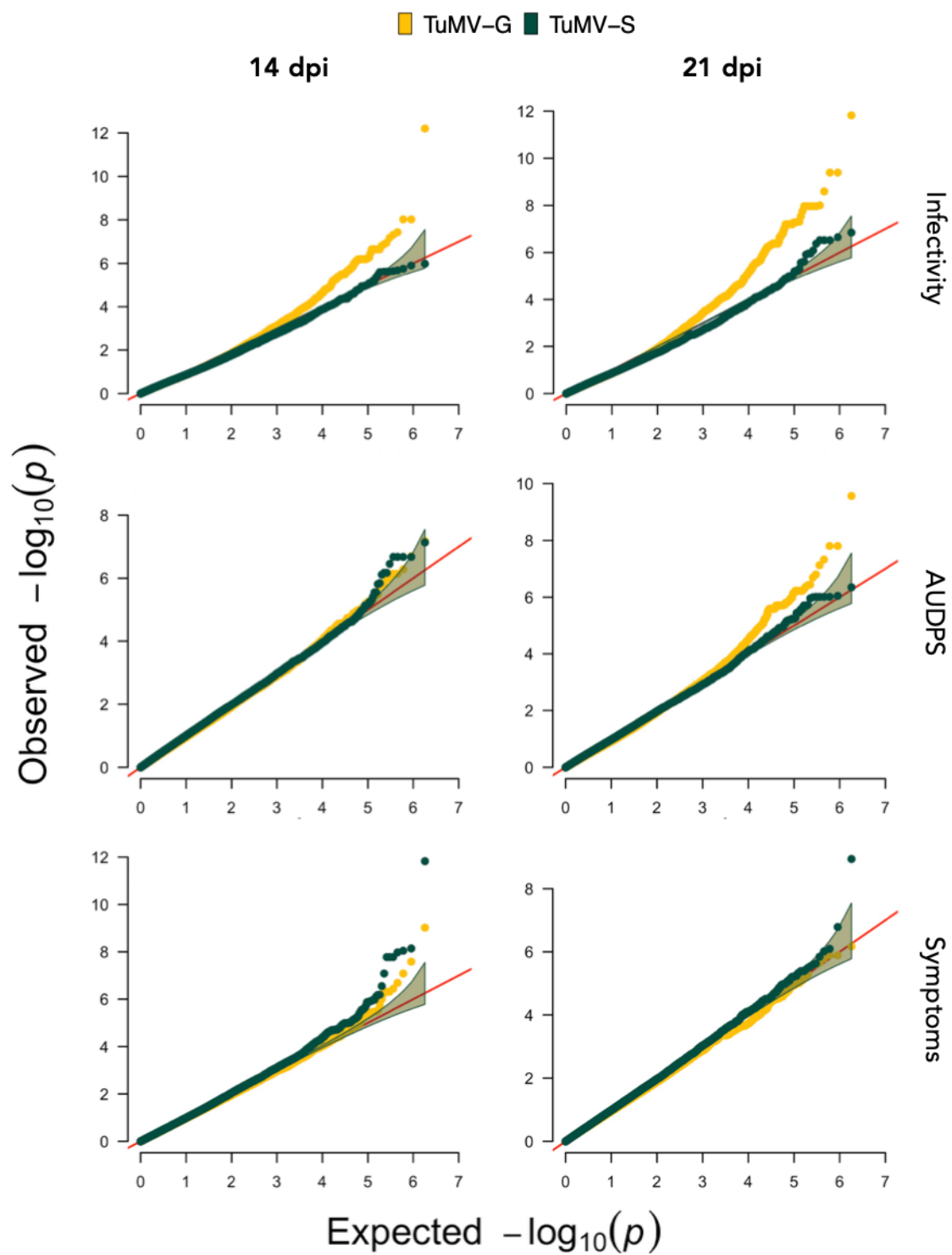

Supplement: veab063_Supp [file veab063_supp.zip › Supplementary Fig. S2.pdf]
